# A Case of Remarkable and Speedy Recovery from Severe Injury of the Spinal Column

**Published:** 1841-11

**Authors:** Solon Borland

**Affiliations:** Memphis, Tenn.


Art. II.—*A Case of remarkable and speedy recovery from severe injury of the Spinal Column.* By Solon Borland, M. D., of Memphis, Tenn.

Believing that, in regard to the importance of the structures involved, the severity of the lesion, and rapidity and completeness of restoration, the following case has no parallel on record; that it demonstrates an amount of strength and elasticity in the *medulla spinalis,* not usually supposed to exist; and, for these reasons, deserves the notice of the profession; I submit an account of it for publication.

Early on Sunday morning, 2nd May last, Dr. James R. Christian called, in haste, at my office, and requested me to visit, with him, a man at the flat-boat landing, who had received a severe injury. We reached him about half an hour after the accident.

*Case.*—Wm. E. Hall, a sailor, aged 20 years, rather below the middle stature, of symmetrical form, stout, muscular, athletic, and in fine health, was seated in a flat-boat, on one of the sills which was about four inches above the level of the floor; his lower limbs were extended and apart, and his body bent forward. The roof of the boat, covered with several tons of wet corn, suddenly fell in, crushing him down, until his face and breast lay pressed upon the floor between his legs. Four or five minutes elapsed before he was extricated.

We found him presenting the symptoms of violent concussion of the brain: entire insensibility, intermittent and slightly stertorous respiration, much froth about the mouth and nostrils, pulse barely perceptible. There was contusion of the nose, and of the upper portion of the back, with extensive and severe ecchymosis of the cheeks, forehead, right side of the neck, and right shoulder; and blood was trickling from the eyes and ears. Proceeding to examine the spine, we found, upon tracing it from its upper and lower extremities, that it was entire, and apparently uninjured, except at the articulation between the *last dorsal* and *first lumbar vertebræ.* The integuments at this point were much contused. The articulation was completely torn asunder, all its ligaments ruptured, the spinous process of the superior vertebra broken off, and the articulating surfaces of the two vertebræ nearly two inches apart; two fingers being easily passed between the vertebræ, without encountering any bony resistance. When the patient was turned on his side, the spinal column formed, at this point of separation, an angular projection of about 100 degrees; the prominence of the dorsal region presenting somewhat the proportion and aspect of a camel's hump. Naturally concluding that the case was beyond the reach of human aid, and that in a few hours, at most, death must close the scene, we expressed that opinion, and retired. Imagine our surprise, when, at the expiration of about three hours, we were again called to the patient, and informed that a change for the better had taken place. Accompanied, now, by Dr. Wyatt Christian, we hastened to the boat. We found the patient partially recovered from the cerebral concussion, with restored sensibility, in great pain, tossing violently from side to side, and moving the lower limbs with some force; his pulse still very frequent, and scarcely less feeble than at our first visit, and his skin rather cool. His eyes, now open, were intensely red—the conjunctiva, like clotted blood, at least three lines in thickness, rising round, and encroaching upon, the cornea. Apprehensive, from present appearances, especially from the movements of the lower limbs, that our previous diagnosis might have been hasty and inaccurate, we now proceeded to make another examination of the spine. Its general aspect was unchanged; and upon a minute, careful, and protracted examination, both the Drs. Christian, and myself, could not hesitate to concur in the declaration—that *the spinous process of the last dorsal vertebra was broken off*,—that *the articulation of that vertebra with the first lumbar was completely sundered, and the articulating surfaces of the two bones nearly two inches apart.* It was now evident, however, that the *medulla spinalis* was not ruptured, as we had, at first, supposed; nor, indeed, materially injured; for both sensation and motion were almost perfect below the point of lesion. In view of all the circumstances, while we admitted their extraordinary nature, and wondered how "the silver cord" had maintained its integrity, it was impossible for us to doubt the accuracy of our present diagnosis; for if it be true, as the ancient adage has it, that "seeing is believing," we felt warranted, by the concurrent evidence of *feeling*, in this case, in according to our impressions the force of positive knowledge. From the extent and severity of the injury our prognosis was still unfavoroble. We gave him ʒi tinct. opi., and directed its repetition if he should be restless at the expiration of an hour; promising to see him again in the evening. During the afternoon, he was carried into town, to more comfortable quarters. The tinct. opi. had been repeated, and he had slept a little. He was then tolerably quiet, but complained of some pain and great thirst; talked a little, but rather incoherently; pulse 125, quick and feeble, but with rather more volume than in the morning; ecchymosis of the face and neck somewhat lessened; eyes still like clots of blood surrounding the deep set cornea; skin cool and moist. Did not examine his spine, as he was lying on it, and suffered great pain on motion. Directed the administration of the opiate, *pro re nata*, until he should be visited again.

May 3rd. Afternoon. Saw him with the Drs. Christian, and Dr. Frayser. Expression of countenance good, ecchymosis very slight, eyes still deeply blood-shot, entire rationality, composed, does not complain of pain except when moved, soreness of all the muscles, moves the lower limbs freely, passed urine this morning, pulse 120 and fuller than on yesterday evening, skin natural. A thorough examination of the spine was not now made, on account of the pain it gave him to be moved and handled. It was found, however, that continued lying on his back, on a hard bed, had nearly straightened the spinal column, and approximated the sundered vertebræ; so that upon the slight examination Dr. Frayser was able to make, he only detected the fracture of the spinous process. The patient was soon placed upon a pallet of quilts on the floor, with instructions to lie constantly on his back.

The treatment, from this time, was quite uniform and simple; consisting of opiate and aperient medicine, *pro re nata*, and a spare farinaceous diet. The patient rapidly improved, suffering little or no febrile reaction, at any time, during his confinement, and sleeping well at night. The ecchymosis, contusion, muscular soreness, and injection of the eyes, speedily disappeared; and all the functions resumed their healthy play. At the end of two weeks he was able to sit up; in a few days more to walk about the room, steadying his steps with a cane; and at the expiration of four weeks he returned, on foot, to the boat, a distance of half a mile.

A few days since, while writing in my office, I heard behind me, the sound of a light, quick, foot-fall. Looking round, I was most agreeably startled at seeing *Hall.* His comely person attired after the neatest summer fashion of a sailor, and his handsome face wreathed in smiles; his eye, now clear and of the brightest blue, sparkling with intelligence, good humor, and perfect health. What a change had come over him! "But yesterday," and he lay at my feet, a crushed and bleeding worm, weltering on the very brink of the grave! Now, he stood before me, erect and sturdy,—as fine a specimen of human organization as the eye of Canova might love to dwell upon. Such, at least, he seemed to my delighted vision.

Only thirty-two days had elapsed since he had received the injury; and he had called to inform me of his entire recovery; and enable me, on a final examination, to close my notes of his case. He bears but slight external traces of the injury: there is a slight displacement, downward, of the fractured spinous process, and inconsiderable thickening, and induration of the muscles and ligaments about the intervertebral articulation which had been so completely severed; but which, now, was to all appearance perfectly approximated, and firmly reunited. Pressure and percussion on the part gave no pain; and the natural power of slight flexion of the spine, in any direction was unimpaired. He had not, of course, yet tried his strength, by lifting, or other violent muscular efforts; and would, prudently, refrain from doing so, for some time to come.

I will not prolong this article by copying the mechanical *rationale*, to which I refer the peculiar form of the lesion, in this case, and which I have appended to my notes. It will naturally suggest itself to any one, who will reflect upon the position Hall occupied at the time of the accident, and the successive changes in that position—presenting different points of contact to the roof of the boat, as the body yielded to its pressure.

So important, do I consider it, to the interest of science, and to the honor and usefulness of the medical profession, that nothing should appear in their annals, as *facts*, which are, merely, believed by those who furnish them; but such only as are fully established; and so extraordinary do I consider this case, that had I been alone in my observations of it, I would not furnish this report, however strict its fidelity to my own impressions. Under circumstances so verging on the marvellous, I would suppress my own unaided conclusions, remain silent, rather than risk, from those who do not know me, the uncharitable imputation of unfaithfulness, or the juster distrust of those who, from a wise and salutary caution, ever question the sufficiency of one witness, whoever he may be, to substantiate an extraordinary occurrence. Sustained, however, as I am, in this case; corroborated as are my views, by those of two other physicians of science and high respectability—one of whom is a practitioner of mature age, enlarged observation, and great experience, I feel warranted in making public that which, to my mind, is as well established as any fact, of the kind, can be by evidence, short of that furnished by dissection.

*Memphis, Tenn., June,* 1841.

We have read the foregoing account of Hall's case, by Dr. Borland, and find it substantially accordant with our own impressions.

W. CHRISTIAN, M. D.
J. R. CHRISTIAN, M. D.

*Memphis, July* 29, 1841.

P. S.—I learn from Dr. J. R. Christian, who has seen the patient several weeks since my last examination, that there is more deformity than I have reported—that there is now considerable backward curvature. This has occurred since I examined the case; and is, doubtless, the result of the upright position, and muscular exertions, which have been too constantly maintained, or sought to be maintained, before the ligaments and muscles had entirely recovered their strength. I cautioned the patient against it. S. B.

